# 2015. Challenges in Assessing Blood Stream Infection Clearance in Patients Receiving Extracorporeal Membrane Oxygenation

**DOI:** 10.1093/ofid/ofac492.1639

**Published:** 2022-12-15

**Authors:** Stone A Frankford, Michal J Sobieszczyk, Ana E Markelz, Joseph E Marcus

**Affiliations:** San Antonio Uniformed Services Health Education Consortium, San Antonio, Texas; Pulmonary/Critical Care - Brooke Army Medical Center, San Antonio, TX, San Antonio, Texas; Brooke Army Medical Center, San Antonio, Texas; Infectious Disease - Brooke Army Medical Center, San Antonio, TX, San Antonio, Texas

## Abstract

**Background:**

There is a limited published data on the treatment of blood stream infections (BSI) in patients receiving extracorporeal membrane oxygenation (ECMO). Traditionally, the management of only fungal and Gram-positive BSIs require follow-up blood cultures to document clearance. The presence of large retained cannulas in ECMO create concern for persistent bacteremia. This study investigates whether certain variables are predictive of blood stream infections with positive repeat cultures (BSIPRC) and if BSIPRC is associated with increased mortality.

**Methods:**

All positive blood cultures from patients receiving ECMO at Brooke Army Medical Center identified between September 2012 and October 2021 were included in this study. Blood cultures were excluded if they were determined to be contaminants by the primary team. For each positive blood culture, charts were retrospectively reviewed. Date of clearance was defined as the first negative blood culture after a positive. BSIPRC was defined as re-isolation of the same organism on repeat blood cultures following an initial positive blood culture.

**Results:**

A total of 60 patients and 87 BSI were investigated (38.5 BSI per 1000 ECMO days). Gram-positive (GP) organisms caused a majority of BSI (n=52, 60%) followed by Gram-negative (GN) (n=22, 25%) and fungal (n=13, 15%) infections. Of the 80 (92%) BSIs who had repeat blood cultures drawn, patients had BSIPRC in 35 (44%) of cases. There were no clinical features that differentiated patients with positive repeat cultures (Table). There was no difference in survival to discharge for patients with BSIPRC as compared to single day BSI (20 (58%) vs. 28 (63%), p=0.78). In 6 patients with BSIPRC with Gram-negative organisms, two (33%) died before clearance and five (83%) died before decannulation.

Characteristics of patients and all blood stream infections.

**Figure:**
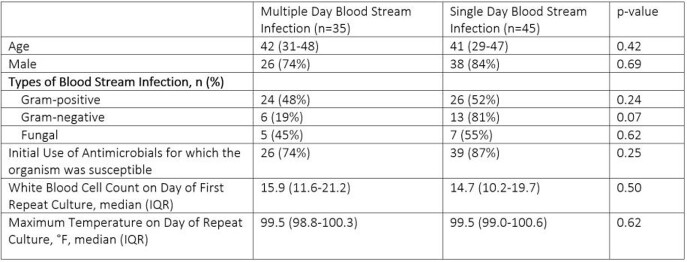

**Conclusion:**

There were no clinical findings that differentiated patients with BSIPRC from those who had a single day of positivity. BSIPRC was associated with high mortality in patients with Gram-negative bacteremia. In the absence of clear predicative factors in demonstrating clearance of BSI, repeat blood cultures may be necessary. Future studies are needed to determine why Gram-negative BSIPRC are associated with a high mortality.

**Disclosures:**

**All Authors**: No reported disclosures.

